# Polyphenols in Liubao Tea Can Prevent CCl_4_-Induced Hepatic Damage in Mice through Its Antioxidant Capacities

**DOI:** 10.3390/nu10091280

**Published:** 2018-09-10

**Authors:** Yanni Pan, Xingyao Long, Ruokun Yi, Xin Zhao

**Affiliations:** 1 Chongqing Collaborative Innovation Center for Functional Food, Chongqing University of Education, Chongqing 400067, China; panyanni@foods.ac.cn (Y.P.); longyaoyao@foods.ac.cn (X.L.); yirk@cque.edu.cn (R.Y.); 2 Chongqing Engineering Research Center of Functional Food, Chongqing University of Education, Chongqing 400067, China; 3 Chongqing Engineering Laboratory for Research and Development of Functional Food, Chongqing University of Education, Chongqing 400067, China; 4 College of Biological and Chemical Engineering, Chongqing University of Education, Chongqing 400067, China; 5 Department of Food Science and Biotechnology, Cha University, Gyeongghi-do 487-010, South Korea

**Keywords:** polyphenol, Liubao tea, hepatic damage, mRNA expression, protein expression

## Abstract

The present study investigated the preventive effect of polyphenols in Liubao tea (PLT) on carbon tetrachloride (CCl_4_)-induced liver injury in mice. The mice were initially treated with PLT, followed by induction of liver injury using 10 mL/kg CCl_4_. Then liver and serum indices, as well as the expression levels of related messenger RNAs (mRNAs) and proteins in liver tissues were measured. The results showed that PLT reduces the liver quality and indices of mice with liver injury. PLT also downregulates aspartate aminotransferase (AST), alanine aminotransferase (ALT), triglycerides (TGs), and malondialdehyde (MDA), and upregulates superoxide dismutase (SOD) and glutathione peroxidase (GSH-Px) in the sera of mice with liver injury. PLT also reduces serum levels of interleukin-6 (IL-6), interleukin-12 (IL-12), tumor necrosis factor-α (TNF-α), and interferon-γ (IFN-γ) cytokines in mice with liver injury. Pathological morphological observation also shows that PLT reduces CCl_4_-induced central venous differentiation of liver tissues and liver cell damage. Furthermore, qPCR and Western blot also confirm that PLT upregulates the mRNA and protein expressions of Gu/Zn-SOD, Mn-SOD, catalase (CAT), GSH-Px, and nuclear factor of κ-light polypeptide gene enhancer in B-cells inhibitor-α (IκB-α) in liver tissues, and downregulates the expression of cyclooxygenase 2 (COX-2) and nuclear factor κ-light-chain-enhancer of activated B cells (NF-κB). Meanwhile, PLT also raised the phosphorylated (p)-NF-κB p65 and cytochrome P450 reductase protein expression in liver injury mice. The components of PLT include gallic acid, catechin, caffeine, epicatechin (EC), epigallocatechin gallate (EGCG), gallocatechin gallate (GCG), and epicatechin gallate (ECG), which possibly have a wide range of biological activities. Thus, PLT imparts preventive effects against CCl_4_-induced liver injury, which is similar to silymarin.

## 1. Introduction

Liubao tea is prepared from Wuzhou large tea leaves from China. It is a specialty tea generated through the processes of natural fermentation, pile fermentation, drying, autoclaving, aging, and other characteristics. Therefore, Liubao tea is a kind of black tea that is post-fermented [1]. This tea is named according to its geographical origin, namely, Liubao County, Wuzhou City, Guangxi Province, China. Liubao tea was historically used as a preventive medicine [2]. The majority of research studies focus on Pu’er tea, Hunan black tea, and Fuzhuan tea, whereas investigations of relevant technology and functions of Liubao tea are limited [3]. Recent studies showed that Liubao tea imparts lipid-lowering effects, regulates glucose and lipid metabolism, possesses anti-oxidation activity, and regulates immune function and intestinal flora. These health benefits come from the components of Liubao tea, which include polyphenols, flavonoids, caffeine, free amino acids, and soluble sugars [4,5,6].

The liver is an important metabolic organ, and damage to this organ can cause severe harm to the human body, which includes liver injury due to chemicals such as higher alcohol intake, drug side effects, and environmental toxic chemicals, ultimately leading to cirrhosis and liver cancer [7]. Carbon tetrachloride (CCl_4_) is a common chemical inducer of liver damage in the laboratory. CCl_4_ triggers the production of high levels of inflammatory cytokines in liver cells during liver injury, which aggravates inflammation and liver damage. Simultaneously, CCl_4_ can induce the formation of Cl^−^ and CCl_3_^−^ in liver cell microsomes, leading to lipid peroxidation of liver microsomes, resulting in lipid peroxidation and destruction of cell membranes, and ultimately, liver damage [8].

Active oxygen free radicals cause oxidative stress, which is a common pathophysiological mechanism of liver diseases. Oxidative stress could cause hepatic damage by inducing membrane lipid peroxidation that changes biofilm function, as well as inducing covalent combinations with biological macromolecules, and destruction of enzyme activities (such as tumor necrosis factor-α (TNF-α) and nuclear factor κ-light-chain-enhancer of activated B cells (NF-κB)) [9]. Oxidative stress plays an important role in fatty liver, viral hepatitis, liver fibrosis, and other liver diseases [10]. Energy metabolism in organisms utilizes oxygen as an electronic acceptor in the process of aerobic metabolism, which inevitably produces reactive oxygen species (ROS). ROS has a dual effect, which is closely related to the regulation of some physiological active substances and the inflammatory immune process, but excessive ROS can easily lead to oxidative stress [11]. The mitochondrial respiratory chain complex uses electron transfer to produce ATP, which is the main source of ROS. The liver is rich in mitochondria, and is, therefore, the main organ susceptible to ROS attack, and oxidative stress has a close relationship with most liver damage [12]. ROS can also initiate a variety of cytokines such as transforming growth factor-beta (TGF-β), interleukin-8 (IL-8), and NF-κB. These cytokines can lead to infiltration of neutrophils, enhance inflammatory response, and ultimately lead to liver cell injury [13].

Tea polyphenols are a very important component of tea. Studies showed that tea polyphenols have a strong scavenging effect on oxygen free radicals [14,15]. Tea polyphenols can sequester lipid peroxidation free radicals during the peroxidation process, lower polyphenolic free-radical content, and interrupt free-radical oxidation chain reactions, thereby effectively removing free radicals [14]. Simultaneously, tea polyphenols can activate and enhance the activity of various antioxidant enzymes such as superoxide dismutase (SOD), glutathione peroxidase (GSH-Px), and catalase (CAT), as well as efficiently eliminate free radicals [16]. Tea polyphenols can prevent lipid peroxidation caused by CCl_4_, as well as avoid the damage of the membrane structure and function of liver cells caused by the covalent binding of CCl_4_ and liver microsomal lipids and proteins [17]. In addition, tea polyphenols impart a protective effect on obstructive jaundice liver injury caused by peroxidation, acute liver injury caused by cadmium poisoning, alcoholic liver injury, and liver cancer [18]. In addition, except for certain reports of catechins and other individual tea polyphenols on liver injury protective effects, the characteristics of the tea polyphenols and polyphenol composition analysis remain unclear, including those of Liubao tea [19].

This study utilized CCl_4_ in establishing a chemical liver injury mouse model to investigate the preventive effect of PLT. We also employed molecular biology methods to test the indices of serum and liver tissue, and the preventive mechanism of PLT on liver injury was elucidated. The results of this study may facilitate the development and utilization of PLT in food processing and the manufacture of health products.

## 2. Materials and Methods 

### 2.1. PLT Extraction

Approximately 150 g of Liubao tea (Guilin Lijiang Tea Factory Co. Ltd., Guilin, Guangxi, China), placed in a 15-L beaker, was mixed with 500 mL of boiling water, extracted in a 95 °C water bath, and after 1 h of filtration, the filtrate was collected. This process was repeated, and the two filtrates were pooled, and thoroughly mixed with 100 g of ZnCl_2_, before the mixture’s pH was adjusted to 7.5 using 2 mol/L ammonia. The mixture was then centrifuged at 3000 rpm for 10 min, and the supernatant was discarded and the precipitate containing the crude polyphenols was collected. Approximately 3 L of hydrochloric acid solution (2 mol/L) was added to the sediment, which was stirred to dissolve. Then, 2 mol/L ammonia was added to adjust the pH of the mixture to 4.0. After filtration, the filtrate was extracted with 4000 mL of ethyl acetate twice, and the extract was evaporated with a rotary evaporator to obtain the polyphenol extract [20].

### 2.2. Experimental Model in Kunming (KM) Mice

The male KM mice (eight weeks old) were divided into five groups (10 mice in each group), which included the normal group, control group, silymarin gavage group (silymarin, positive control group), low-dose PLT group via intragastric administration (LPLT group, 50 mg/kg), and high-dose PLT group via intragastric administration (HPLT group, 100 mg/kg). The mice were first allowed to acclimatize to the laboratory conditions for one week. The mice in the normal group and the control group were intragastrically given 2 mL of normal saline. The mice in the silymarin-instilled group were given 0.2 mL of silymarin solution daily at a dose of 100 mg/kg. The high-/low-dose PLT group mice were treated with PLT at 100 mg/kg and 50 mg/kg, respectively, daily for two weeks. All mice except for the normal group were intraperitoneally injected with CCl_4_ inducer (2 mL/kg; CCl_4_: olive oil = 1:1, *v*/*v*) on the 14th day, and then all mice were fasted, but allowed to drink water. The mice were sacrificed after fasting for 24 h, and their hearts were collected for dissection, whereas the livers were isolated for later use [21]. The liver index was calculated using the formula as liver weight/body weight × 100. This study was approved by the Animal Ethics Committee of Chongqing University of Education (Chongqing, China).

### 2.3. Measurement of Serum Indices

The mouse serum samples were separated by centrifugation at 4000 rpm for 10 min. The serum levels of aspartate aminotransferase (AST), alanine aminotransferase (ALT), triglycerides (TGs), SOD, GSH-Px, and malondialdehyde (MDA) were determined using assay kits (Nanjing Jiancheng Bioengineering Institute, Nanjing City, China).

### 2.4. Cytokine Levels in Serum

The serum samples were isolated by centrifugation and cytokine levels were assayed using IL-6 (ab46100), IL-12 (ab119531), TNF-α (ab100747), and interferon-γ (IFN-γ; ab100689) cytokine assay kits (Abcam, Cambridge, MA, USA).

### 2.5. Histopathological Analysis of Liver Tissues

Mouse liver tissue samples were fixed in 10% formalin solution for 24 h, followed by dehydration in 95% ethanol for 24 h. Then, the tissues were sectioned, stained with hematoxylin and eosin (H&E), and then assessed under a BX43 microscope (Olympus, Tokyo, Japan). The grading of liver injury is shown in Table 1. 

### 2.6. qPCR Analysis

The messenger RNA (mRNA) expression of liver tissue in mice was determined by SYBR green assay. The liver tissues were homogenized, followed by total RNA extraction using TRIzol reagent (Thermo Fisher Scientific, Waltham, MA, USA). The concentration of RNA was detected using a micro-ultraviolet (UV) spectrophotometer (Nano 300, Aosheng, Hanzhou, Zhejiang, China). Approximately 1 μg of mRNA was reverse transcribed into complementary DNA (cDNA). The PCR conditions were as follows: pre-denaturation at 95 °C for 3 min, followed by 40 cycles of denaturation at 95 °C for 10 s, annealing at 57 °C for 30 s, and extension at 72 °C for 15 s [22]. The primers in this study are shown in Table 2. The relative transcription levels of the mRNAs were calculated using the 2^−ΔΔCr^ method.

### 2.7. Western Blot Analysis

The 100-mg liver tissue samples were homogenized with 1 mL of radio immunoprecipitation assay (RIPA) and 10 µL of phenylmethanesulfonyl fluoride (PMSF); then, they were centrifuged at 12,000 rpm (5 min, 4 °C), the hepatocytes were lysed, and the lysate was kept on ice for 30 min. The bicinchoninic acid (BCA) method was used to determine the protein concentration. The sample was mixed with an equal volume of 5× loading buffer and then placed in a water bath at 100 °C for 5 min. The hepatocytes were then sonicated by SJIALAB for 2 min (10% ultrasound intensity), and centrifuged (4500 rpm) for 10 min to separate the supernatant. The extracted protein was then subjected to polyacrylamide gel electrophoresis (80–120 V), and transferred onto a polyvinylidene fluoride (PVDF) membrane, sealed, and incubated overnight at 4 °C with the corresponding primary antibodies, namely, cyclooxygenase 2 (COX-2; MA514568, Thermo Fisher Scientific), inducible nitric oxide synthase (iNOS) (PA1036), NF-κB (PA1186), phosphorylated (p)-NF-κB p65 (MA515160), nuclear factor of κ-light polypeptide gene enhancer in B-cells inhibitor-α (IκB-α; 397700), Cu/Zn-SOD (PA5270240), Mn-SOD (PA530604), GSH-Px (PA540504), CAT (PA259183), cytochrome P450 reductase (PA577820), and β-actin (MA5157739), incubated at 37 °C with the second antibody (A21241) for 1 h, followed by colorimetric detection and chemiluminescence imaging (iBright FL1000, Thermo Fisher Scientific) [23].

### 2.8. High-Performance Liquid Chromatography (HPLC) Assay

The standard products of gallic acid, catechin, caffeine, epicatechin (EC), epigallocatechin gallate (EGCG), gallocatechin gallate (GCG), and epicatechin gallate (ECG) were weighed accurately, and the standard products were placed in 50-mL volumetric flasks, to the appropriate amount of methanol. The mixture was vortexed to dissolve, and diluted to scale with methanol, i.e., to obtain the standard stock solution. One milliliter of gallic acid, catechin, caffeine, EC, EGCG, GCG, and ECG stock solutions were each placed into 10-mL volumetric flasks and mixed with an equal volume of methanol to obtain a mixed standard solution. The PLT extract was extracted with precision, and a 0.5 mg/mL solution was prepared using methanol. PLT components were detected (UltiMate3000 HPLC System, Thermo Fisher Scientific) using the following chromatographic conditions: Accucore perfluorophenyl (PFP) column (4.6 mm × 150 mm, 2.6 μm, Thermo Fisher Scientific); flow rate of 0.6 mL/min; detection wavelength of 280 nm; injection volume of 10 L; column temperature of 30 °C; collection time of 20 min; and mobile phases A for acetonitrile, and B for 0.1% formic acid solution. The gradient elution conditions are shown in Table 3 [24].

### 2.9. Statistical Analysis

The data are expressed as the mean ± standard deviation (SD). Differences between mean values for each group were assessed by one-way ANOVA with Duncan’s new multiple-range test (MRT). Differences with a *p* < 0.05 were considered statistically significant. The SAS v9.1 statistical software package (SAS Institute, Cary, NC, USA) was used for these analyses.

## 3. Results

### 3.1. Body Weight, Liver Weight, and Liver Indices of the Experimental Mice

As shown in Table 4, on the first day, there was no significant difference (*p* > 0.05) in body weight across all mice. On the 14th day, the body weight of the control group was significantly higher (*p* < 0.05) than that of the other groups because of individual differences, while the mice in the Liupao tea treatment group had lower body weight gain than that of the other groups probably because of the lipid-reducing effect of Liupao tea. After being treated with CCl_4_, the body weight of mice in the control group was the heaviest, whereas that of the other groups was lower than that of the control mice. The liver weight and liver indices of mice in control group were also the highest, while the liver weight and liver indices of mice in the normal group were the lowest. Because of the treatment with PLT, the liver indices of the hepatic damage-induced mice decreased compared to those of the control group, and the HPLT group exhibited lower liver indices than the LPLT group. The indices of the HPLT group were also roughly similar to the silymarin group.

### 3.2. Serum AST, ALT, and TG Levels

Table 5 shows that the serum AST, ALT, and TG levels of mice in the normal group were the lowest, whereas those of the control group were the highest. The serum AST, ALT, and TG serum levels of mice in the HPLT group were significantly higher (*p* < 0.05) than those of the silymarin group, but were significantly lower (*p* < 0.05) than those of the LPLT group.

### 3.3. Serum SOD, GSH-Px, and MDA Levels

Table 6 shows that the serum SOD and GSH-Px levels of mice in the normal group were the highest, whereas the SOD and GSH-Px levels of those in the HPLT group were significantly higher (*p* < 0.05) than those in the LPLT and control groups, but lower than those in the silymarin group. However, the MDA levels in mice of the HPLT group were significantly lower (*p* < 0.05) than those of the LPLT and control groups, but significantly higher (*p* < 0.05) than those of the silymarin and normal groups.

### 3.4. Serum IL-6, IL-12, TNF-α, and IFN-γ Cytokine Levels

Table 7 shows that the serum IL-6, IL-12, TNF-α, and IFN-γ cytokine levels of mice in the normal group were lowest; these levels were lower than those of the silymarin, HPLT, LPLT, and control groups in decreasing order.

### 3.5. Histopathological Assessment of the Liver

**Figure 1:**
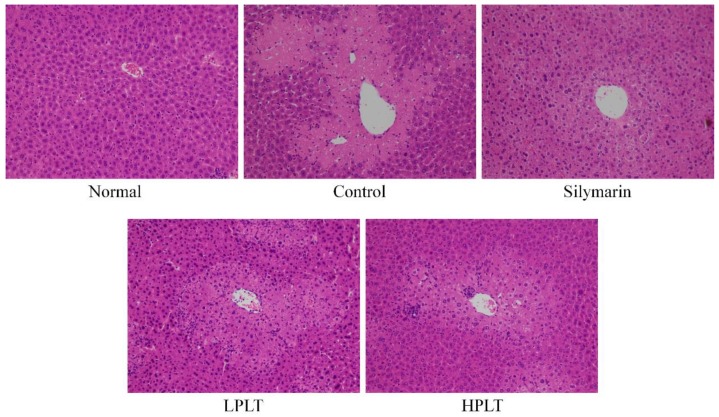
Hematoxylin and eosin (H&E) pathological observation of hepatic tissue in experimental mice with CCl_4_-induced hepatic damage. Magnification: 100×. Silymarin group: 50 mg/kg body weight (b.w.) silymarin treatment dose; LPLT group: 50 mg/kg b.w. polyphenols of Liubao tea (PLT) low (L) treatment dose; HPLT group: 100 mg/kg b.w. polyphenols of Liubao tea (PLT) high (H) treatment dose.

CCl_4_ induced liver injury in mice of the control, silymarin, LPLT, and HPLT groups; the observed histopathological changes included degeneration and necrosis of the centrilobular cells. Normally, the central veins of the liver are rounded, the hepatocytes are uniform in size, and they are evenly arranged in a radial pattern around the central veins. The liver tissues of mice in the control group showed the most severe damage (Grade 4, Figure 1). Silymarin (Grade 1) and PLT reduced these hepatic injury changes, and silymarin facilitated the reduction in damage incurred by the hepatic tissues. HPLT (Grade 2) imparted effects similar to that observed with silymarin, and only a few liver cells demonstrated hemorrhage in the area around the centrilobular vein.

### 3.6. mRNA and Protein Expression of Cu/Zn-SOD, Mn-SOD, GSH-Px, and CAT in Mouse Hepatic Tissues

**Figure 2:**
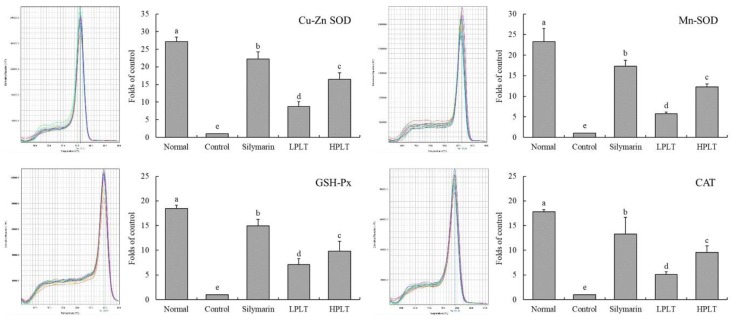
Cu/Zn- superoxide dismutase (SOD), Mn-SOD, glutathione peroxidase (GSH-Px), and catalase (CAT) messenger RNA (mRNA) expressions in hepatic tissue of experimental mice with CCl_4_-induced hepatic damage. Values presented are the means ± standard deviation (*N* = 3/group). ^a–e^ Mean values with different letters in the same bars are significantly different (*p* < 0.05) and those with the same letter in the same column are not significantly different (*p* > 0.05) according to Duncan’s new multiple-range test (MRT). Silymarin group: 50 mg/kg b.w. silymarin treatment dose; LPLT group: 50 mg/kg b.w. polyphenols of Liubao tea (PLT) treatment dose; and HPLT group: 100 mg/kg b.w. polyphenols of Liubao tea (PLT) treatment dose.

**Figure 3:**
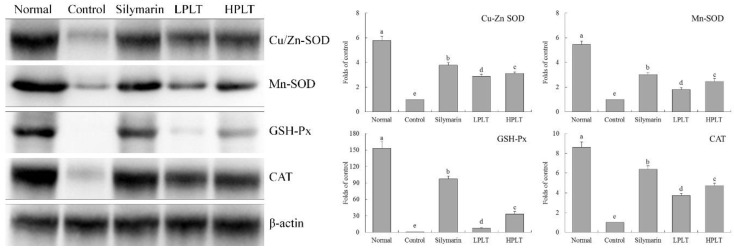
Cu/Zn-SOD, Mn-SOD, GSH-Px, and CAT protein expressions in hepatic tissue of experimental mice with CCl_4_-induced hepatic damage. Values presented are the means ± standard deviation (*N* = 3/group). ^a–e^ Mean values with different letters in the same bars are significantly different (*p* < 0.05) and those with the same letter in the same column are not significantly different (*p* > 0.05) according to Duncan’s new MRT. Silymarin group: 50 mg/kg b.w. silymarin treatment dose; LPLT group: 50 mg/kg b.w. polyphenols of Liubao tea (PLT) treatment dose; and HPLT group: 100 mg/kg b.w. polyphenols of Liubao tea (PLT) treatment dose.

The mRNA and protein expressions of Cu/Zn-SOD (27.13-fold (mRNA) and 5.79-fold (protein) increase relative to control group), Mn-SOD (23.30-fold (mRNA) and 5.47-fold (protein) increase relative to control group), GSH-Px (18.43-fold (mRNA) and 153.10-fold (protein) increase relative to control group), and CAT (17.80-fold (mRNA) and 8.63-fold (protein) increase relative to control group) in the hepatic tissues of mice in the normal group were the highest, whereas those of the control group were the lowest (Figure 2 and Figure 3). After treatment with silymarin and PLT, the Cu/Zn-SOD, Mn-SOD, GSH-Px, and CAT expressions of liver in CCl_4_-treated mice were reduced; the HPLT-treated mice exhibited higher Cu/Zn-SOD (16.46-fold (mRNA) and 3.11-fold (protein) increase relative to control group), Mn-SOD (13.31-fold (mRNA) and 2.47-fold (protein) increase relative to control group), GSH-Px (9.85-fold (mRNA) and 33.52-fold (protein) increase relative to control group), and CAT (9.53-fold (mRNA) and 4.74-fold (protein) increase relative to control group) expressions than the LPLT-treated mice (8.71-, 5.71-, 7.11-, and 5.10-fold (mRNA) and 2.87-, 1.81-, 7.52-, and 3.74-fold (protein) increase, respectively, relative to control group), but weaker than the silymarin-treated (22.18-, 17.33-, 14.92-, and 13.27-fold (mRNA) and 3.78-, 3.02-, 97.47-, and 6.40-fold (protein) increase, respectively, relative to control group) mice.

### 3.7. mRNA and Protein Expression of COX-2, iNOS, NF-κB, and IκB-α in Mouse Hepatic Tissues

**Figure 4:**
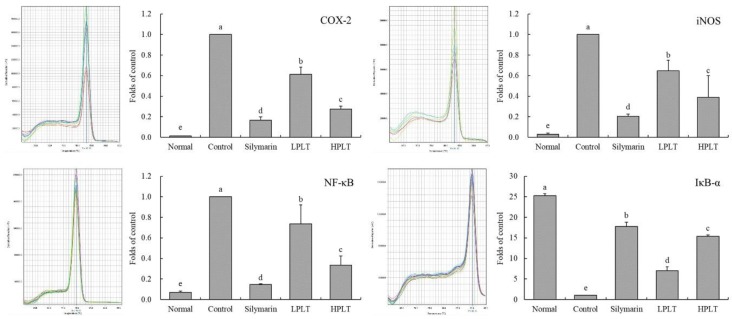
Cyclooxygenase 2 (COX-2), inducible nitric oxide synthase (iNOS), nuclear factor κ-light-chain-enhancer of activated B cells (NF-κB), and nuclear factor of κ-light polypeptide gene enhancer in B-cells inhibitor-α (IκB-α) mRNA expressions in hepatic tissue of experimental mice with CCl_4_-induced hepatic damage. Values presented are the means ± standard deviation (*N* = 3/group). ^a–e^ Mean values with different letters in the same bars are significantly different (*p* < 0.05) and those with the same letter in the same column are not significantly different (*p* > 0.05) according to Duncan’s new MRT. Silymarin group: 50 mg/kg b.w. silymarin treatment dose; LPLT group: 50 mg/kg b.w. polyphenols of Liubao tea (PLT) treatment dose; and HPLT group: 100 mg/kg b.w. polyphenols of Liubao tea (PLT) treatment dose.

**Figure 5:**
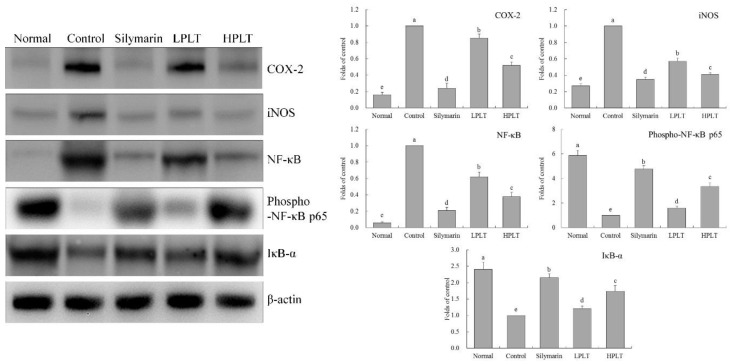
COX-2, iNOS, NF-κB, phosphorylated (p)-NF-κB p65, and IκB-α protein expressions in hepatic tissue of experimental mice with CCl_4_-induced hepatic damage. Values presented are the means ± standard deviation (*N* = 3/group). ^a–e^ Mean values with different letters in the same bars are significantly different (*p* < 0.05) and those with the same letter in the same column are not significantly different (*p* > 0.05) according to Duncan’s new MRT. Silymarin group: 50 mg/kg b.w. silymarin treatment dose; LPLT group: 50 mg/kg b.w. polyphenols of Liubao tea (PLT) treatment dose; and HPLT group: 100 mg/kg b.w. polyphenols of Liubao tea (PLT) treatment dose.

The IκB-α mRNA (25.30-fold increase relative to control group) and protein (2.41-fold increase relative to control group) expressions of mice in the normal group were the highest, whereas the COX-2 (0.01-fold (mRNA) and 0.16-fold (protein) increase relative to control group), iNOS (0.03-fold (mRNA) and 0.27-fold (protein) increase relative to control group), and NF-κB (0.07-fold (mRNA) and 0.06-fold (protein) increase relative to control group) expressions in the normal group were the lowest (Figure 4 and Figure 5). The IκB-α mRNA (15.38-fold increase relative to control group) and protein (1.74-fold increase relative to control group) expressions of mice in the HPLT group were only lower than those of the silymarin (17.80-fold (mRNA) and 2.15-fold (protein) increase relative to control group) and normal groups, but stronger than those of the LPLT (7.03-fold (mRNA) and 1.21-fold (protein) increase relative to control group) and control groups. The HPLT group showed lower COX-2 (0.28-fold (mRNA) and 0.52-fold (protein) increase relative to control group), iNOS (0.39-fold (mRNA) and 0.41-fold (protein) increase relative to control group), and NF-κB (0.33-fold (mRNA) and 0.38-fold (protein) increase relative to control group) expression levels than the LPLT (0.61-, 0.65-, and 0.74-fold (mRNA) and 0.85-, 0.57-, and 0.62-fold (protein) increase, respectively, relative to control group) and control groups, but only slightly stronger than the silymarin group (0.17-, 0.20-, and 0.15-fold (mRNA) and 0.24-, 0.35-, and 0.21-fold (protein) increase, respectively, relative to control group). Meanwhile, mice in the normal group showed the strongest p-NF-κB p65 protein expression (5.88-fold increase relative to control group), while silymarin-treated mice also showed stronger p-NF-κB p65 protein expression (4.74-fold increase relative to control group) than that of the LPLT- (1.59-fold increase relative to control group) and HPLT- (3.35-fold increase relative to control group) treated mice.

### 3.8. Protein Expression of Cytochrome P450 Reductase in Mouse Hepatic Tissues

**Figure 6:**
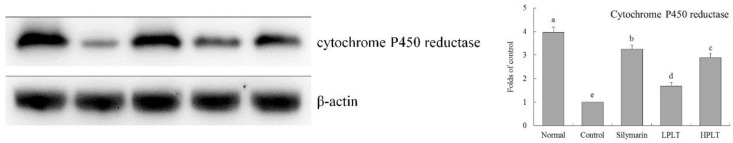
Cytochrome P450 reductase protein expression in hepatic tissue of experimental mice with CCl_4_-induced hepatic damage. Values presented are the means ± standard deviation (*N* = 3/group). ^a–e^ Mean values with different letters in the same bars are significantly different (*p* < 0.05) and those with the same letter in the same column are not significantly different (*p* > 0.05) according to Duncan’s new MRT. Silymarin group: 50 mg/kg b.w. silymarin treatment dose; LPLT group: 50 mg/kg b.w. polyphenols of Liubao tea (PLT) treatment dose; and HPLT group: 100 mg/kg b.w. polyphenols of Liubao tea (PLT) treatment dose.

The cytochrome P450 reductase protein (3.97-fold increase relative to control group) expression of mice in the normal group was highest (Figure 6), and HPLT-treated mice (2.8-fold increase relative to control group) also showed a higher expression than that of LPLT-treated mice (1.69-fold increase relative to control group), but it was lower than that of silymarin-treated mice (3.25-fold increase relative to control group).

### 3.9. Constituents of PTL

**Figure 7:**
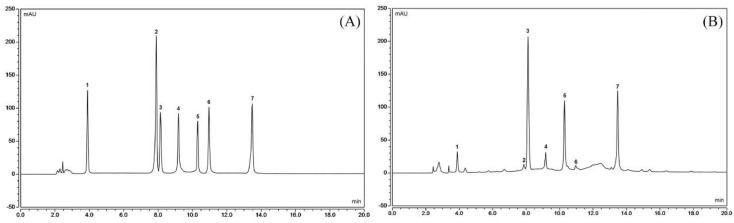
Polyphenol constituents of Liubao tea (PLT). (**A**) Standard chromatograms; (**B**) PLT chromatograms. 1: gallic acid; 2: catechin; 3: caffeine; 4: epicatechin (EC); 5: epigallocatechin gallate (EGCG); 6: gallocatechin gallate (GCG); 7: epicatechin gallate (ECG).

Figure 7 shows that PTL contains seven kinds of polyphenols, namely, gallic acid, catechin, caffeine, EC, EGCG, GCG, and ECG, with contents of 4.98%, 4.20%, 16.71%, 0.90%, 7.29%, 3.03%, and 34.44%, respectively.

## 4. Discussion

Liubao tea is a non-toxic food and meets the requirements of food safety according to the standards of food toxicology [25]. Liupao tea was shown to have weight-loss effects [3], and Liupao tea polyphenols were also found to inhibit weight gain in mice in this study. Therefore, there were individual differences in the body weight of mice before carbon-tetrachloride (CTC) treatment. Liver injuries may result in harmful and sometimes life-threatening effects to the body. Liver quality and liver index, which are indices of CTC-induced liver injury, were used in the present study [26]. The results show that PLT can reduce the liver quality and liver indices of mice with liver injury, and these effects are similar to those using the liver injury drug, silymarin.

ALT and AST are expressed by hepatocytes; ALT is secreted into the cytoplasm, whereas AST is mainly produced in the mitochondria of hepatocytes. Damage to cells due to hepatitis, myocarditis, and pancreatitis induces ALT to enter the bloodstream. However, during severe damage, AST also enters the bloodstream [27]. Thus, a significant increase in ALT and AST levels indicates liver damage [28]. Liver injury can lead to the transfer of fatty acids to the liver, resulting in increased intrahepatic TG content, and TG levels also reflect the degree of liver lipid peroxidation [29]. In this study, PLT was found to inhibit the increase in ALT, AST, and TG levels caused by carbon tetrachloride liver injury. ALT, AST, and TG are the most typical clinical liver function indicators [27,28]. Observing the influence of these indicators can judge whether the liver function is normal or not. It could be seen that PLT had a certain effective role in restoring normal liver function.

CCl_4_ will lead to the body’s oxidation; the body utilizes two defenses, namely, non-enzymatic and enzymatic, to prevent oxidative damage, including regulation of SOD, CAT, and GSH-Px, which are the main mechanisms for enzymatic oxidation [30]. SOD catalyzes superoxide radicals and is capable of scavenging free radicals, whereas CAT and SOD synergistically enhance the role of free radicals [31]. GSH-Px is an important enzyme that catalyzes the decomposition of hydrogen peroxide, which in turn, protects cell membranes and prevents cell damage [32]. MDA is a metabolite of lipid peroxidation; a high content of MDA accumulates in the body after liver injury [33]. In this study, we found that PLT could significantly regulate the levels of SOD, GSH-Px, and MDA in the body caused by liver injury, thereby protecting the liver from the effects of carbon tetrachloride.

CCl_4_ induces oxidization and liver inflammation, resulting in a significant increase in serum IL-6, IL-12, TNF-α, and IFN-γ levels in mice [21]. IL-6 is a factor secreted by T helper 2 (Th2) cells and is involved in the humoral immune response. An increase in Th2 levels may result in visceral dysfunction [34]. IL-6 promotes the differentiation, proliferation, and antibody production of T lymphocytes. It can also change intracellular G cell activity and upregulate neutrophil function, as well as enhance inflammatory reactions of the body [35]. IL-12 is an activating factor of natural killer (NK) cells, and its effect is the most intense. High rates of apoptosis in hepatocytes and excessive immune response during liver injury further aggravate the condition, which is related to the fact that IL-12 increases the cytotoxicity of cluster of differentiation 8 (CD8)^+^ T cells [36]. Binding of TNF-α and liver cell membrane TNF-α receptor 1 (TNF-αR1) can induce intracellular double-stranded DNA to fragment, thereby resulting in stem cell apoptosis. In addition, TNF-α triggers inflammatory responses by activating NF-κB, which exacerbates liver injury [37]. IFN-γ is a proinflammatory cytokine that increases the sensitivity of hepatocytes to TNF-α, rendering hepatocytes to further damage [38]. Oxidative stress after liver tissue damage can cause an imbalance in the level of inflammatory cytokines such as TNF-α, IL-1β, and IL-6, which increases in the levels of TNF-α, IL-1β, and IL-6 in the liver [39]. Through the detection of inflammatory cytokines, we also found that PLT could inhibit inflammation by reducing the level of inflammatory factors, thereby reducing liver injury.

Mn-SOD and Gu/Zn-SOD are SOD isomers [40]. Mn-SOD is an SOD radical scavenger in the mitochondria [41]. Gu/Zn-SOD is an SOD free radical scavenger in the cytoplasm and takes Cu^2+^ and Zn^2+^ as its active center [42]. The liver and heart are organs that are rich in mitochondria, and Mn-SOD activity markedly decreases after CCl_4_-induced liver injury [43]. The same result was obtained in this study. Gu/Zn-SOD can purify the toxic effects of O^2−^ in the body, and protect the visceral tissues [44]. Studies showed that CCl_4_ causes oxidative stress reactions in the body, resulting in the excessive production of free radicals. Mn-SOD and Gu/Zn-SOD can inhibit free radicals in the body, and play a preventive role in liver injury [45,46]. CAT is an important antioxidant enzyme in the body. CAT can eliminate H_2_O_2_ in the body, thereby inhibiting oxidative stress, reducing the body’s oxidation caused by carbon tetrachloride, and inhibiting liver injury [47]. Through the detection of gene and protein expression, it was further found that PLT could regulate the expressions of oxidation-related proteins in tissues, thereby reducing the damage caused by oxidative stress to tissues, thus protecting the liver.

NF-κB is a key factor in the regulation of inflammatory response, including inflammation-related IL-6 and TNF-α; these are upregulated during inflammation. Under normal circumstances, NF-κB and IκB-α in the bound state show an inactivation of both. NF-κB and IκB-α in extrinsic inflammatory conditions lead to the inhibition of inflammation by binding to IκB-α to activate NF-κB [48]. Meanwhile, through phosphorylation of NF-κB, it can reduce the promotion of inflammation of NF-κB and alleviate tissue damage [49]. NO is a highly active oxidant that is produced in the liver by activated NOS in liver cells, and promotes the high expression of the *iNOS* gene upon liver damage progression. In liver injury, oxidative stress occurs in hepatocytes, and a large number of inflammatory factors are released [50]. iNOS is an important inflammatory factor, and iNOS is very active in inflammation. iNOS-induced NO also promotes further damage to the liver [51]. COX-2 is also an important inflammatory factor; the tissue is not expressed under normal conditions. COX-2 expression raises after liver injury, whereby Kupffer cells are activated, and COX-2 upregulation exacerbates the liver inflammation [52]. Further experiments showed that PLT could regulate the expressions of COX-2, iNOS, NF-κB, p-NF-κB p65, and IκB-α, alleviating the liver injury caused by inflammation and carbon tetrachloride.

Most foreign compounds depend on metabolism by P450 in the liver. When carbon tetrachloride induces acute liver injury, lipid peroxidation and a large number of free radicals are produced in the liver, resulting in a decrease in activity of cytochrome P450. At the same time, carbon tetrachloride directly inhibits the synthesis of the cytochrome P450 enzyme, and the decrease in cytochrome P450 enzyme activity directly or indirectly leads to a decrease in detoxification ability of the liver, thereby aggravating liver injury [53]. Upon carbon tetrachloride treatment, PLT could raise the activity of cytochrome P450 reductase, and inhibited the liver injury.

Gallic acid (GA) can inhibit oxidative stress and cytotoxicity to improve liver injury. The activation of hepatic stellate cells (HSCs) caused by liver injury is an important part of liver fibrosis [54]. GA can also induce HSCs to produce O^2−^, OH^−^, and H_2_O_2_, thereby inducing oxidative stress that selectively kills HSCs [55]. Catechin also affects free-radical scavenging by reducing the content of MDA and increasing the activity of SOD [56]. Catechin has inhibitory effects on chronic hepatitis [57]. IL-1β can induce hepatic acute-phase protein synthesis, thereby affecting normal liver activity [58], while caffeine suppresses the production of inflammatory molecules, thus preventing the activating of the immune system in IL-1β [59], which may play a role in liver protection. Animal model studies showed that caffeine could also inhibit acute alcoholic liver injury possibly by imparting antioxidant effects and inhibiting the expressions of IL-1β and TNF-α [60]. EC also has antioxidant effects that influence cardiovascular disease, hypertension, cancer, and obesity [61]. EC could reduce the inflammatory-related expression of NF-κB, iNOS, and TNF-α [62]. Oxidative stress is considered to be the main cause of CCl_4_-induced liver damage. CCl_4_ is metabolized by cytochrome P450 in hepatocytes to produce three chloromethyl radicals. These radicals cause lipid peroxidation and lipid peroxidation products, which cause liver cell damage and promote the formation of fibrous tissue. EGCG plays a role in anti-CCl_4_-induced liver fibrosis in rats through its antioxidant capacity [63]. The effects of EGCG on anti-liver injury are also reflected in the inhibition of TNF-α and IFN-γ by EGCG expression, thus preventing further immune damage caused by TNF-α and IFN-γ [64]. GCG can inhibit oxidative damage to tissues and protect viscera from oxidative damage [65]. ECG has anti-cancer effects, possibly stronger than EGCG [66]. ECG has better melanin inhibition effects than EGCG, and ECG shows antioxidant effects, which are greater than EGCG [67]. These active components are combined together to form PLT, thereby strongly inhibiting liver injury. PLT is a mixture with substantial biological activity. Its action may be the combined action of many substances, and its specific mechanism needs further study.

In this study, toxic carbon tetrachloride was used to simulate chemical-induced liver injury, and the observed effects remained at the laboratory level. In order to better prove this study’s argument, future research on the human body is expected. In addition, the role of PLT in liver injury needs to be further studied, which will be conducive to more obvious discoveries of the link between its active components and their mechanisms. At the same time, in view of the mechanism of PLT, it is necessary to verify the mechanism more accurately for the differences across PLT components in the future.

## 5. Conclusions

This study induced hepatic injury in mice, and the hepatic injury-reducing effects of PLT were determined. PLT reduced the liver weight and liver indices in hepatic injury mice. PLT also reduced serum AST, ALT, TG, and MDA levels and increased serum SOD and GSH-Px levels in hepatic injury mice. Meanwhile, PLT reduced serum IL-6, IL-12, TNF-α, and IFN-γ cytokine levels in mice with hepatic injury. Further investigation showed that PLT upregulates the mRNA and protein expressions of Mn-SOD, Gu/Zn-SOD, CAT, GSH-Px, and IκB-α, and downregulates COX-2, iNOS, and NF-κB in mice with hepatic injury. PLT contains gallic acid, catechin, caffeine, EC, EGCG, GCG, and ECG, and its effects are similar to the drug silymarin. PLT is a functional ingredient that may be used as a raw material in functional foods.

## Figures and Tables

**Table 1:** Pathological grading of liver injury.

Grade	Portal Area and Surrounding Area	Hepatic Lobule
0	No inflammation	No inflammation
1	Portal inflammation	Degeneration and few necrotic foci
2	Mild detrital necrosis	Degeneration, focal necrosis
3	Moderate detrital necrosis	Degeneration or necrosis, or bridge necrosis
4	Severe detrital necrosis	Bridge necrosis wide range, involving multiple lobules, leaflet structure disorder

**Table 2:** qPCR assay sequences.

Gene	Forward Sequence	Reverse Sequence
*COX-2*	5′–GGTGCCTGGTCTGATGATG–3′	5′–TGCTGGTTTGGAATAGTTGCT–3′
*iNOS*	5′–GTTCTCAGCCCAACAATACAAGA–3′	5′–GTGGACGGGTCGATGTCAC–3
*NF-κB*	5′–ATGGCAGACGATGATCCCTAC–3′	5′–CGGAATCGAAATCCCCTCTGTT–3′
*IκB-α*	5′–TGAAGGACGAGGAGTACGAGC–3′	5′–TGCAGGAACGAGTCTCCGT–3′
*Cu/Zn-OD*	5′–AACCAGTTGTGTTGTCAGGAC–3′	5′–CCACCATGTTTCTTAGAGTGAGG–3′
*Mn-SOD*	5′–CAGACCTGCCTTACGACTATGG–3′	5′–CTCGGTGGCGTTGAGATTGTT–3′
*GSH-Px*	5′–CCACCGTGTATGCCTTCTCC–3′	5′–AGAGAGACGCGACATTCTCAAT–3′
*CAT*	5′–GGAGGCGGGAACCCAATAG–3′	5′–GTGTGCCATCTCGTCAGTGAA–3′
*GAPDH*	5′–AGGTCGGTGTGAACGGATTTG–3′	5′–GGGGTCGTTGATGGCAACA–3′

**Table 3:** Flow phase gradient elution program.

t/min	A/%	B/%
0	10	90
6.5	18.5	81.5
20	29.5	70.5

**Table 4:** Body weight, liver weight, and liver indices in experimental mice with CCl_4_-induced hepatic damage.

Group	1st Day Body Weight (g)	14th Day Body Weight (g)	15th Day Body Weight (g)	Liver Weight (g)	Liver Index
Normal	35.17 ± 0.32 ^a^	42.66 ± 1.59 ^b^	42.12 ± 1.96 ^b^	1.57 ± 0.07 ^b^	3.73 ± 0.18 ^e^
Control	35.53 ± 0.28 ^a^	48.22 ± 2.62 ^a^	46.64 ± 1.38 ^a^	2.41 ± 0.18 ^a^	5.15 ± 0.23 ^a^
Silymarin	35.06 ± 0.22 ^a^	41.43 ± 0.55 ^b^	40.35 ± 0.84 ^b^	1.58 ± 0.08 ^b^	3.92 ± 0.19 ^d^
LPLT	35.19 ± 0.26 ^a^	36.62 ± 1.45 ^c^	35.61 ± 1.81 ^c^	1.61 ± 0.12 ^b^	4.51 ± 0.11 ^b^
HPLT	35.41 ± 0.20 ^a^	36.63 ± 2.41 ^c^	35.84 ± 3.70 ^c^	1.48 ± 0.15 ^b^	4.14 ± 0.03 ^c^

Values presented are the means ± standard deviation (*N* = 10/group). ^a–e^ Mean values with different letters in the same column are significantly different (*p* < 0.05) and those with the same letter in the same column are not significantly different (*p* > 0.05) according to Duncan’s new multiple-range test (MRT). Silymarin group: 50 mg/kg body weight (b.w.) silymarin treatment dose; LPLT group: 50 mg/kg b.w. polyphenols of Liubao tea (PLT) low (L) treatment dose; HPLT group: 100 mg/kg b.w. polyphenols of Liubao tea (PLT) high (H) treatment dose.

**Table 5:** Serum aspartate aminotransferase (AST), alanine aminotransferase (ALT), and triglyceride (TG) levels in experimental mice with CCl_4_-induced hepatic damage.

Group	AST (U/L)	ALT (U/L)	TG (pg/mL)
Normal	6.20 ± 0.43 ^e^	1.54 ± 0.22 ^e^	150.00 ± 26.15 ^e^
Control	21.13 ± 0.93 ^a^	17.98 ± 1.53 ^a^	563.75 ± 16.18 ^a^
Silymarin	12.13 ± 0.35 ^d^	4.09 ± 0.44 ^d^	208.75 ± 20.06 ^d^
LPLT	17.75 ± 0.57 ^b^	12.36 ± 2.23 ^b^	385.00 ± 57.72 ^b^
HPLT	13.85 ± 0.55 ^c^	8.45 ± 0.64 ^c^	273.75 ± 32.89 ^c^

Values presented are the means ± standard deviation (*N* = 10/group). ^a–e^ Mean values with different letters in the same column are significantly different (*p* < 0.05) and those with the same letter in the same column are not significantly different (*p* > 0.05) according to Duncan’s new MRT. Silymarin group: 50 mg/kg b.w. silymarin treatment dose; LPLT group: 50 mg/kg b.w. polyphenols of Liubao tea (PLT) low (L) treatment dose; and HPLT group: 100 mg/kg b.w. polyphenols of Liubao tea (PLT) high (H) treatment dose.

**Table 6:** Serum superoxide dismutase (SOD), glutathione peroxidase (GSH-Px), and malondialdehyde (MDA) levels in experimental mice with CCl_4_-induced hepatic damage.

Group	SOD (U/mL)	GSH-Px (U/mL)	MDA (nmol/mL)
Normal	121.38 ± 4.88 ^a^	85.92 ± 1.83 ^a^	2.24 ± 0.06 ^e^
Control	58.56 ± 2.42 ^d^	5.52 ± 1.02 ^e^	5.93 ± 0.45 ^a^
Silymarin	107.11 ± 1.77 ^b^	63.94 ± 3.20 ^b^	2.89 ± 0.16 ^d^
LPLT	78.44 ± 8.35 ^c^	25.35 ± 1.03 ^d^	4.44 ± 0.21 ^b^
HPLT	106.12 ± 1.37 ^b^	53.49 ± 2.84 ^c^	3.34 ± 0.26 ^c^

Values presented are the means ± standard deviation (*N* = 10/group). ^a–e^ Mean values with different letters in the same column are significantly different (*p* < 0.05) and those with the same letter in the same column are not significantly different (*p* > 0.05) according to Duncan’s new MRT. Silymarin group: 50 mg/kg b.w. silymarin treatment dose; LPLT group: 50 mg/kg b.w. polyphenols of Liubao tea (PLT) low (L) treatment dose; and HPLT group: 100 mg/kg b.w. polyphenols of Liubao tea (PLT) high (H) treatment dose.

**Table 7:** Cytokine interleukin (IL)-6, IL-12, tumor necrosis factor-α (TNF-α), and interferon-γ (IFN-γ) levels in experimental mice with CCl_4_-induced hepatic damage.

Group	IL-6 (pg/mL)	IL-12 (pg/mL)	TNF-α (pg/mL)	IFN-γ (pg/mL)
Normal	31.11 ± 1.84 ^d^	26.17 ± 3.06 ^d^	365.40 ± 16.75 ^e^	32.32 ± 0.59 ^d^
Control	64.33 ± 3.80 ^a^	56.68 ± 6.98 ^a^	718.76 ± 40.28 ^a^	77.94 ± 1.60 ^a^
Silymarin	41.02 ± 3.01 ^c^	34.50 ± 1.90 ^c^	467.22 ± 38.47 ^d^	39.07 ± 0.27 ^c^
LPLT	54.27 ± 6.05 ^b^	42.46 ± 4.92 ^b^	622.90 ± 50.68 ^b^	44.61 ± 0.79 ^b^
HPLT	45.19 ± 1.24 ^c^	36.69 ± 0.55 ^bc^	547.63 ± 26.83 ^c^	39.10 ± 0.56 ^c^

Values presented are the means ± standard deviation (*N* = 10/group). ^a–e^ Mean values with different letters in the same column are significantly different (*p* < 0.05) and those with the same letter in the same column are not significantly different (*p* > 0.05) according to Duncan’s new MRT. Silymarin group: 50 mg/kg b.w. silymarin treatment dose; LPLT group: 50 mg/kg b.w. polyphenols of Liubao tea (PLT) low (L) treatment dose; and HPLT group: 100 mg/kg b.w. polyphenols of Liubao tea (PLT) high (H) treatment dose.
